# Apoptosis in mouse fetal and neonatal oocytes during meiotic prophase one

**DOI:** 10.1186/1471-213X-7-87

**Published:** 2007-07-24

**Authors:** Fataneh Ghafari, Carlos G Gutierrez, Geraldine M Hartshorne

**Affiliations:** 1 Department of Biological Sciences, University of Warwick, Coventry, CV4 7AL, UK; 2 Facultad de Medicina Veterinaria, Universidad Nacional Autonoma de Mexico, DF 04510, Mexico; 3 Clinical Sciences Research Institute, Warwick Medical School, University of Warwick, Coventry, CV2 2DX, UK; 4 Centre for Reproductive Medicine, University Hospitals Coventry and Warwickshire NHS Trust, Coventry, CV2 2DX, UK

## Abstract

**Background:**

The vast majority of oocytes formed in the fetal ovary do not survive beyond birth. Possible reasons for their loss include the elimination of non-viable genetic constitutions arising through meiosis, however, the precise relationship between meiotic stages and prenatal apoptosis of oocytes remains elusive. We studied oocytes in mouse fetal and neonatal ovaries, 14.5–21 days post coitum, to examine the relationship between oocyte development and programmed cell death during meiotic prophase I.

**Results:**

Microspreads of fetal and neonatal ovarian cells underwent immunocytochemistry for meiosis- and apoptosis-related markers. COR-1 (meiosis-specific) highlighted axial elements of the synaptonemal complex and allowed definitive identification of the stages of meiotic prophase I. Labelling for cleaved poly-(ADP-ribose) polymerase (PARP-1), an inactivated DNA repair protein, indicated apoptosis. The same oocytes were then labelled for DNA double strand breaks (DSBs) using TUNEL. 1960 oocytes produced analysable results.

Oocytes at all stages of meiotic prophase I stained for cleaved PARP-1 and/or TUNEL, or neither. Oocytes with fragmented (19.8%) or compressed (21.2%) axial elements showed slight but significant differences in staining for cleaved PARP-1 and TUNEL to those with intact elements. However, fragmentation of axial elements alone was not a good indicator of cell demise. Cleaved PARP-1 and TUNEL staining were not necessarily coincident, showing that TUNEL is not a reliable marker of apoptosis in oocytes.

**Conclusion:**

Our data indicate that apoptosis can occur throughout meiotic prophase I in mouse fetal and early postnatal oocytes, with greatest incidence at the diplotene stage. Careful selection of appropriate markers for oocyte apoptosis is essential.

## Background

The aim of this study was to identify and quantify apoptosis at different stages of meiotic prophase I in mouse oocytes, in order to explore the relationship between chromosomal activity during meiosis, and the occurrence of cell death by apoptosis.

Most mammalian oocytes die long before they reach maturity, having no direct role in forming the next generation. Extensive loss of immature oocytes occurs at various stages in mice: (1) during meiotic prophase I, the prenatal process of oocyte formation; (2) in the first days after birth when oocytes that have not been enclosed into primordial follicles suffer demise and (3) when the ovarian follicle that nurtures the oocyte succumbs to atresia. Follicular recruitment, growth and atresia are tightly controlled by intra-ovarian factors and gonadotrophic hormones. However, the factors balancing oocyte formation and loss prenatally have received less attention, even though these are crucial for establishing the size and quality of the ovarian reserve.

The biological basis for the prenatal cull of oocytes remains unexplained. For example, it may be a developmental solution to accumulated mutations in mitochondria [1], a means of avoiding inheritance of potentially lethal errors arising during germ cell mitosis or meiotic prophase I [2], or an altruistic process ensuring survival of some oocytes within a particular sibling 'nest' [3].

While oocyte populations behave predictably, the factors controlling survival or death of individual oocytes remain obscure. Synaptic problems are common and may promote oocyte loss [4] while defects in recombination caused by DNA repair insufficiency can trigger meiotic arrest [5]. Thus, selective elimination based on meiotic abnormality could promote the survival of more normal oocytes to the ovarian pool [6]. However, these quality control mechanisms are not completely efficient, allowing some abnormal oocytes to continue developing. In humans, mature oocytes have an exceptionally high rate of around 20% aneuploidy [7]. Such aneuploidies may have their origin in meiotic prophase I and are recognised contributors to the low fertility of humans, the high miscarriage rate, and certain prevalent conditions such as Trisomy 21 Down's Syndrome [8]. An understanding of the origins of abnormal oocytes, and the biological methods for their control, has potential to improve reproductive outcome. We are therefore interested in how abnormalities in oocytes during meiotic prophase I relate to the occurrence of apoptosis. These experiments in mice complement and extend our studies of human prenatal oogenesis [9-11].

In mice, early studies indicated that cell death affects proliferating primordial germ cells or oogonia in 12–13 dpc ovaries, and also oocytes at the zygotene/pachytene stage of meiotic prophase I, from 16 dpc through to birth [reviewed in [12]]. In humans, oocyte loss has been reported particularly at the pachytene stage, using electron microscopic identification of meiotic chromosomes [2]. Prenatal loss of oocytes may involve apoptosis [13,14] although this view has been challenged [15]. Several approaches have been made to characterise apoptotic oocytes in mouse fetal ovaries. Small oocytes with reduced DNA content were observed at 13.5 dpc [16] and increased on 15.5 and 17.5 dpc [17], DNA ladders (180–200 bp) have been detected by gel electrophoresis, and DNA fragmentation in oocytes has been detected by TUNEL applied to ovarian tissue sections [18]. The germ cell specific marker Vasa, has been applied together with poly (ADP-ribose) polymerase (PARP-1) and TUNEL as apoptotic markers [3]. The latter used ovarian tissue sections to show that mouse germ cell apoptosis occurs predominantly from 20.5 to 22.5 dpc when oocytes are mainly in the diplotene stage. Previous publications therefore differ in their interpretation of the risks of oocyte death by apoptosis during the stages of meiotic prophase I.

The study of apoptosis in oocytes is challenging for two reasons. First, DNA breaks, often used as a marker of apoptosis, are integral to meiosis, particularly during the leptotene stage, when DNA strands condense before synapsis [19]. Hence, methods detecting DNA breaks, such as TUNEL, must be combined with apoptosis-specific markers, in order to avoid false positive results [20]. Second, identification of stages of meiotic prophase I has not been straightforward. Histological methods permit differences in interpretation, causing widespread variations in results, notable in studies of human fetal ovaries [reviewed in [21]]. More recently, the availability of molecular methods to identify meiotic chromosomes categorically [22] and to ascribe their meiotic stages with certainty in large numbers of individual cells has prompted us to re-examine the timing of apoptosis in relation to meiotic prophase I.

## Results and Discussion

**Figure 1:**
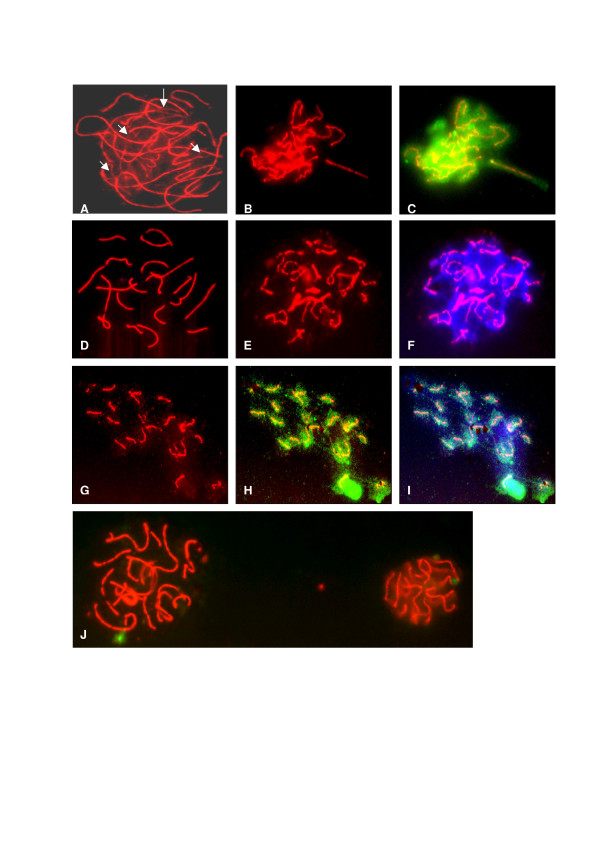
**Microspread oocytes from fetal mouse ovaries, demonstrating different appearances of axial elements and labelling for cleaved PARP-1 and TUNEL during meiotic prophase I**. Red = COR1 immunodetection indicating the axial elements of oocyte chromosomes, used to determine the stage of meiotic prophase I. Blue = Cleaved PARP-1 immunodetection. Presence of cleaved PARP-1 indicates inability to repair DNA damage, indicative of apoptosis. Green = TUNEL labelling. Indicates presence of DNA double strand breaks. A. Oocyte in late zygotene with intact axial elements shown by COR1 staining in red. This oocytes was negative for both cleaved PARP-1 and TUNEL. B and C. Oocyte at pachytene showing discontinuities in COR1 staining. This oocyte was TUNEL positive (green) and negative for cleaved PARP-1. D. Oocyte in pachytene with intact axial elements, negative for both cleaved PARP-1 and TUNEL. E and F. Oocyte in early diplotene showing discontinuities in COR1 staining. This oocyte was positive for cleaved PARP-1 (blue) and negative for TUNEL. G, H and I. Oocyte with short sections of dense discontinuous COR1 staining, possibly degenerating diplotene stage. This oocyte stained positive for both TUNEL (H) and cleaved PARP-1 (I), indicating advanced apoptosis. J. Two adjacent oocytes stained for COR1, demonstrating clear differences in nuclear size. Oocytes showing limited expansion, such as that on the right, we have termed 'compressed'. Both of these oocytes were TUNEL negative (shown green) and PARP-1 positive (not shown).

We studied a total of 1960 oocytes from 24 mouse fetuses or neonates from 14.5 to 21 dpc. The relationship between oocyte development and death during meiotic prophase I was investigated according to the stage of meiotic prophase I, the appearance of axial elements and also their cleaved PARP-1 and TUNEL labelling. Figure 1 presents examples of labelled spread oocytes.

### Progression of oocytes during meiotic prophase I

**Figure 2:**
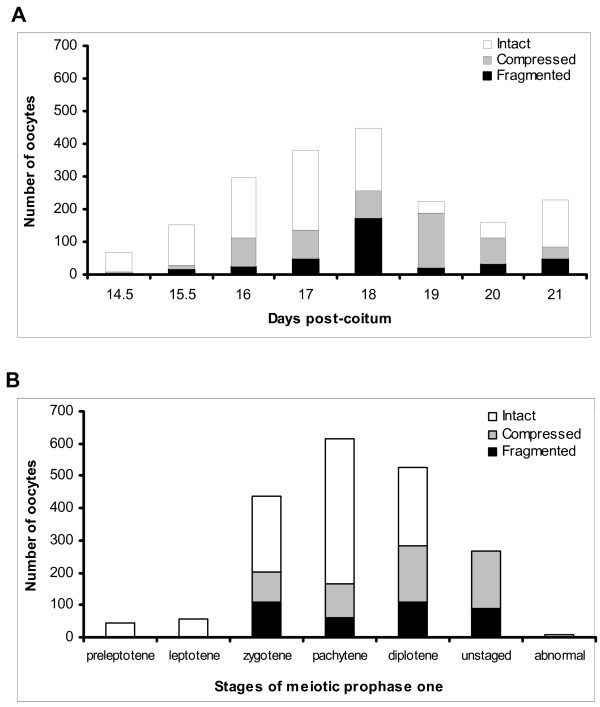
**Total numbers of oocytes identified in mouse fetal and neonatal ovaries, having intact, compressed or fragmented axial elements**. A. on each day post-coitum between 14.5 and 21. B. at each stage of meiotic prophase I.

Overall, the numbers of oocytes in meiotic prophase I, observed using COR1 labelling, increased from 14.5 to 18 dpc and approximately halved at day 19, the day of birth in these mice. Figure 2 shows the numbers of oocytes observed according to age, and stage of meiotic prophase I, indicating the proportions having intact, compressed or fragmented elements. The numbers of oocytes observed are not necessarily representative of the total in ovaries since we only observed those oocytes that remained affixed to slides after microspreading, fixation and washing. However, we are not aware of any evidence that the attachment of chromosomes is biased towards any particular type of cell.

Oocytes with fragmented axial elements comprised 10–25% of zygotene, pachytene and diplotene oocytes, and 33% of unstaged oocytes. Some fragmented oocytes were noted each day from 14.5 to 21 dpc, however they were particularly evident on day 18 (38%) (Figure 2a). Compressed oocytes (see Figure 1J) comprised 17–35% of oocytes at zygotene to diplotene stages, and 65% of unstaged oocytes (Figure 2b). Compressed oocytes were the predominant fraction on day 19 when they comprised 75% of the total. As will be discussed later, the lack of spreading (thus compressed) could be related to compromised membrane function in the degenerating oocytes. Therefore, compared to spread oocytes, compressed oocytes may represent cells with lower viability. Nine oocytes were classed as abnormal.

**Figure 3:**
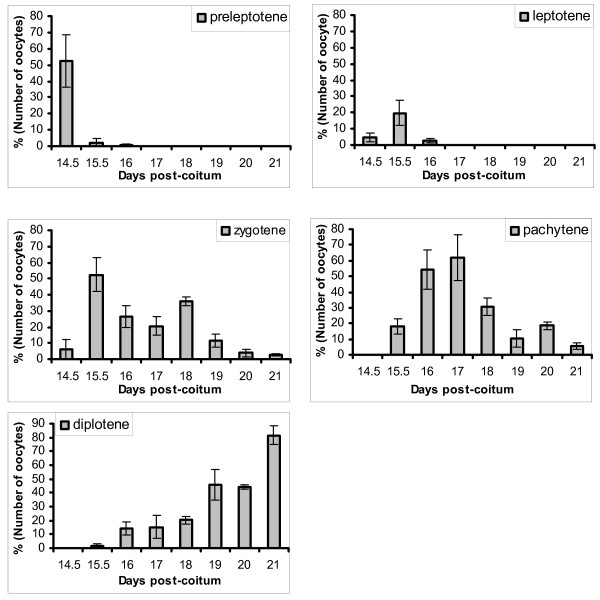
**The proportions of fetal mouse oocytes in different stages of meiotic prophase I between 14.5 and 21 days post coitum**. Note, two waves of zygotene peaking on 15.5 and 18 dpc, followed by two waves of pachytene on 17 and 20 dpc. Note also the persistence of some pre-diplotene stages of oocytes until day 21, two days after birth.

The distribution of oocytes across the stages of meiotic prophase I varied with age as expected (p < 0.001). Unstaged oocytes were notable mainly after 18 dpc. Interestingly, there were two waves of zygotene oocytes on 15.5 and 18 dpc and pachytene oocytes on 16–17 and 20 dpc (Figure 3).

### Factors affecting oocyte labelling with cleaved PARP-1 and TUNEL

**Figure 4:**
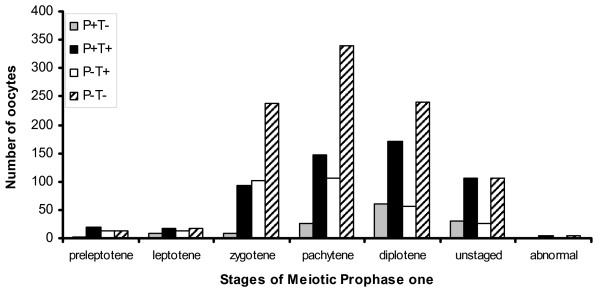
**Distribution of oocytes in the stages of meiotic prophase I according to their labelling for cleaved PARP-1 and/or TUNEL**. P^+ ^indicates positive staining for cleaved PARP-1; T^+ ^indicates positive labelling using the TUNEL method

**Figure 5:**
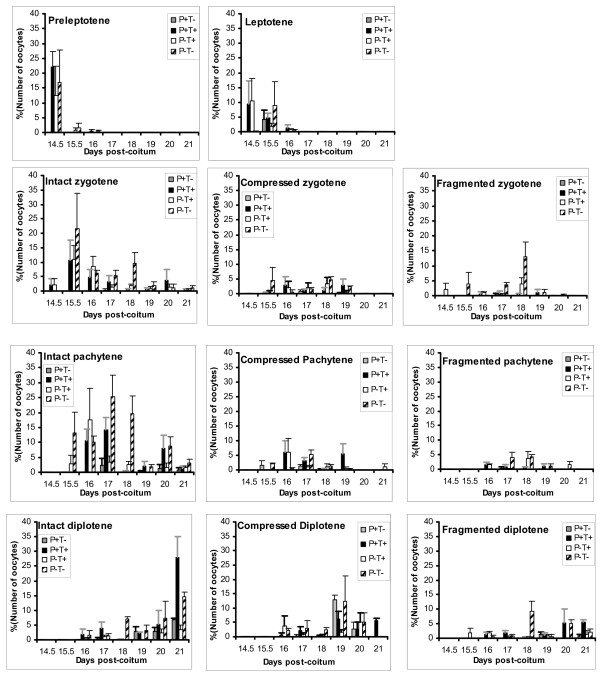
**Distribution of oocytes at each stage of meiotic prophase I between 14.5 and 21 dpc according to their labelling for cleaved PARP-1 and/or TUNEL**. Oocytes with intact axial elements between zygotene and diplotene stages are presented in separate graphs (left) in parallel with oocytes having compressed or fragmented axial elements at the same stage (right).

The oocytes in each category of cleaved PARP-1 and TUNEL labelling (P^+^T^+^, P^+^T^-^, P^-^T^+^, P^-^T^-^) were analysed as a proportion of the total numbers of oocytes observed on the slide of the same ovary. The majority of P^+ ^oocytes were also T^+^, as expected from the relationship between cleaved PARP-1 and DNA damage during apoptosis, however, isolated T+ labelling of oocytes was also evident, as we had predicted. Labelling for cleaved PARP-1 and TUNEL varied according to the stage of meiotic prophase I and age post-coitum (p < 0.001) (Figures 4 and 5). Oocytes staining for neither cleaved PARP-1 nor TUNEL were the largest fraction at all stages of meiotic prophase I.

The data were then analysed according to stage of meiotic prophase I on certain dpc, to determine whether oocytes entering meiosis earlier or later than average were more prone to apoptosis, as has been suggested by Park and Taketo [23].

At the leptotene stage, T^+ ^oocytes predominated on 14.5 dpc (Figure 5), as might be expected from the chromosomal reorganisation taking place in early meiosis [19]. However, by 15.5 dpc, leptotene oocytes appeared to be either apoptotic (P^+^) or potentially viable (P^-^T^-^) (figure 5).

At subsequent stages of development, oocytes exhibited all possible combinations of PARP-1 and TUNEL staining, irrespective of whether their axial elements, highlighted by COR1, appeared intact or fragmented. There was no evidence to suggest that oocytes that entered meiosis earlier or later had different profiles of staining for TUNEL and cleaved PARP-1. It is notable that a few zygotene and pachytene oocytes that were apparently viable (P^-^T^-^) and had intact elements, were identified up to two days after birth (Figure 5). On 20 dpc, 12.9% of oocytes were classified as intact pachytene cells, reducing to 7.4% on 21 dpc. Interestingly, diplotene oocytes increased as expected towards the time of birth. However, at day 21, the principal group of diplotene oocytes was undergoing apoptosis, as shown by P^+^T^+ ^staining (Figure 5).

Statistical analysis of data pooled across time points showed that oocytes at the diplotene stage had a significantly different staining profile to those at pachytene or zygotene stages, regardless of whether their elements were intact or fragmented, having a higher likelihood of P^+^T^+ ^labelling (p < 0.01). At the zygotene, pachytene and diplotene stages, oocytes with fragmented axial elements were less likely to show T^+ ^or P^+ ^labelling than those with intact axial elements (p < 0.01). Additionally, oocytes having compressed chromosomes were significantly more likely to stain for cleaved PARP-1 and TUNEL than those with intact or fragmented axial elements (p < 0.001). Zygotene and pachytene oocytes with compressed elements were also more likely to be P^+^T^- ^than if their elements were intact or fragmented (p < 0.05). A similar relationship was not found for the unstaged oocytes.

A number of questions arising from the data presented have bearing upon the detection of normality and viability in fetal oocytes, and upon our understanding of the processes governing prenatal oocyte selection. These will be highlighted below.

### Can the appearance of synaptonemal complex staining define oocyte viability?

Since a large proportion of prenatal oocytes will die, we wished to understand, firstly, whether oocytes in meiotic prophase I could be ascribed reliably as viable or non-viable on the basis of the visual appearance of their immunostained axial elements. There is considerable discrepancy in the literature concerning the interpretation of microscopic images of meiotic cells as well as the optimal means of cell preparation to obtain ideal cytogenetic spreads. For example, non-intact elements observed microscopically have been interpreted as abnormal, non-viable or degenerating [11,19,24-29] as well as potentially artifactual [30,31].

We found that oocytes with fragmented elements were a relatively consistent proportion of the total at all stages of meiotic prophase I (Figure 2b), averaging 18.5%. This was similar to the 14.8% noted by Martinez-Flores et al [31] in rats using procedures optimised to minimise artifactual fragmentation. The reason for the increased fragmentation on day 18 is unclear. Technical variation has been discounted because mice from different litters gave the same results. Oocytes with fragmented axial elements were less likely to stain for cleaved PARP-1 and TUNEL. Although this finding was significant, its impact was modest because such oocytes represented a relatively small proportion of the total. Overall, staining of oocytes with fragmented axial elements was broadly similar to those with intact axial elements, i.e. the majority remaining unstained for cleaved PARP-1 and TUNEL, despite fragmented axial elements. We therefore conclude that fragmentation of axial elements observed through immunocytochemistry of COR1 is not indicative of a PARP-1 dependent apoptotic process of oocyte degeneration. Nevertheless, fragmentation of SCs is associated with abnormal oogenesis in some mutant mice [32]. Therefore, its association with non-apoptotic degeneration, or apoptosis via a pathway independent of PARP-1, cannot be excluded.

### Cytogenetic spreading favours viable cells

We also explored whether cytogenetic spreads of fetal ovaries produce preparations that are representative of the prenatal oocyte population. We examined all oocytes identified by COR1 staining, regardless of spread appearance, because we thought that the spreading method may selectively prepare viable cells. Spreading includes incubation in hypotonic solution to promote membrane rupture and efficient removal of cytoplasm [33], which may thereby cause under-representation of oocytes in apoptosis or with poor membrane function. Our findings support this contention. The oocytes we observed with compressed elements had nuclei that spread less (Figure 1J) and were also more likely to stain for cleaved PARP-1, a marker of apoptosis. Coucouvanis et al. [16] have also reported condensed nuclei as features of apoptotic oocytes. Such oocytes are a major population at meiotic prophase I (28% in our series), although they are unlikely to contribute to the ovarian reserve. They may therefore have been overlooked in studies where only oocytes producing good quality spreads were analysed. We therefore decided to include these compressed oocytes because their exclusion, based on their suboptimal response to the spreading technique, may be a misrepresentation of the dynamics of cell death within the fetal ovary.

Interestingly, we noted an increase in such compressed oocytes on day 19, soon after birth, heralding the dramatic reduction of oocyte numbers known to occur from birth [3]. The high proportion of P^+^T^+ ^diplotene oocytes at day 21 may be a later manifestation of this phenomenon of postnatal oocyte death.

### Progress of meiotic prophase I

The increasing numbers of oocytes observed between 14.5 and 18 dpc and the profile of stages of meiotic prophase I suggest that female germ cells are entering meiosis gradually, as expected [34], notwithstanding technical losses of cells during processing. Data from McClellan et al. [35] using CD1 mice show a similar profile of stages of meiotic prophase I, although they did not observe diplotene oocytes until the day of birth (also 19 dpc), whereas we observed them in substantial numbers from 16 dpc (Figure 3). The rate of progress of meiotic prophase I has been reported to differ in different strains of mice [34] which may account for this difference. The time profile of meiotic prophase I in our study, showed two 'waves' of zygotene oocytes on 15.5 and 18 dpc and two 'waves' of pachytene oocytes on 16–17 and 20 dpc (Figure 3). The existence of two waves of pachytene oocytes in B6CBF2 mice is consistent with other data from our group (unpublished) and could be a genetic effect [36,37]. Biphasic observations of prenatal oocyte degeneration and the premeiotic S phase have been reported in rats [30,38].

Meiotic prophase I in female mice is non-synchronous and probably takes about four days. The durations of the leptotene, zygotene and pachytene stages in mice were estimated at 3–8 hours, 12–40 hours and >60 hours respectively by Crone et al [39] using tritium labelling studies. Our data show some discrepancies from this approximation, as follows: First, the interval between the first appearance of pachytene at 15.5 dpc and diplotene at 16 dpc is shorter than the expected >60 h duration reported by Crone et al [39]. Our data may suggest either a shorter pachytene stage or a difference in the interpretation of the onset of diplotene, perhaps caused by technical differences between the immunocytogenetic spreads that we used and the autoradiographed sections used by Crone et al [39]. Second, zygotene oocytes remain a major fraction until 19 dpc, while leptotene oocytes are not seen beyond 16 dpc (Figure 3). This 3 day interval is longer than the estimated maximum of 40 hours from Crone et al. [39], suggesting a prolonged zygotene stage in at least some oocytes, probably including the 'second wave' zygotene oocytes that we observed on 18 dpc (Figure 3) and potentially also the pre-diplotene oocytes remaining after birth. Prolonged early meiosis has been associated with persistence of the bouquet stage consequent upon compromised DNA repair, essential for recombination [see [40]]. It may be hypothesised that constraints upon the progression of meiosis, such as the necessity for DNA repair, difficulties with homologous chromosome identification or pairing, or other undefined problems, may indicate an oocyte that is abnormal or has reduced gametogenic potential. The major reduction in zygotene and pachytene cells around birth, whether or not mediated via apoptosis, may thus constitute a selection mechanism against developmentally incompetent oocytes that have failed to reach the diplotene stage and accrete a follicle. If this idea is correct, this mechanism might explain some of the variability in oocyte apoptosis, described in the next section, rather than the pachytene arrest that is well known in males [41].

The persistence of some zygotene and intact pachytene oocytes between 19 and 21 dpc, without indications of apoptosis, has not previously been reported. This finding confirms earlier histological and electron microscopic observations on newborn mice [34,42] but is contrary to the report of McClellan et al [35] in CD1 mice. McClellan et al [35] detected early diplotene oocytes after birth, but zygotene oocytes were absent and pachytene oocytes represented <1% of the total. Recent contributions have revisited the idea that oocyte production may continue into maturity through the persistence of non-meiotic germinal stem cells after birth [43], however, the postnatal longevity of oocytes in prediplotene stages of meiotic prophase I is unknown and further data are required.

### Apoptosis detection in oocytes

Molecular localisation of apoptotic and meiotic markers in microspread oocyte nuclei offers a powerful tool to unravel the inter-related processes of meiosis and apoptosis through detailed analysis of many individual oocytes.

When we studied oocytes using cleaved PARP-1 and TUNEL labelling, both P^+^T^+ ^and P^-^T^- ^germ cells were identified at the preleptotene stage. Positivity for cleaved PARP-1 indicates that some germ cells may be lost through apoptosis even as they enter meiosis. This agrees with previous findings that cell death affects proliferating primordial germ cells or oogonia as well as oocytes at the zygotene, pachytene and/or diplotene stages [13,14].

The high proportion of P^+^T^+ ^cells at the preleptotene and leptotene stages on 14.5 dpc (Figure 5) are a consequence of apoptosis, whereas those with isolated T^+ ^labelling probably have meiotic double strand breaks (DSBs). DSBs appear early in meiotic prophase I (leptotene), prior to the formation of mature SCP3, and disappear in zygotene as synapsis progresses [44,45]. Active RNA synthesis can also result in TUNEL positivity in tissue sections [20]. While RNA synthesis may occur during all stages of meiotic prophase I excepting the pachytene stage and particularly at the diplotene stage [46], the spreading methods we used are likely to have removed this confounding influence. Consistent with this, we did not observe a stage-related incidence of isolated T^+ ^labelling.

Interestingly, we found that TUNEL did not highlight all leptotene oocytes. There may be a number of explanations, for example, that TUNEL does not label meiotic DSBs efficiently, that leptotene oocytes are heterogeneous, that the number of DSBs at the leptotene stage is smaller than the number of 3'-ends in DNA during apoptosis and thus DSBs in leptotene oocytes may be below the threshold for detection by TUNEL, or that the complexes of proteins that bind to meiotic DSBs have persisted despite proteinase K exposure and masked the sites. Further experimentation would be necessary to clarify this point, including the use of antibodies specific to meiotic DSB processing proteins [see [47]], rather than the non-specific TUNEL procedure.

### When does oocyte apoptosis occur?

The proportion of P^+^T^- ^oocytes remained very low throughout meiotic prophase I. This would be expected because cleaved PARP-1 is present late in apoptosis shortly before DNA breakdown, which would then be indicated by co-positivity for TUNEL. However, the observed P^+^T^- ^oocytes in pachytene on 17 dpc and diplotene oocytes from 19 dpc may be precursors of the rise in P^+^T^+ ^diplotene oocytes between 19 and 21 dpc (figures 5). This tends to confirm the findings of Pepling and Spradling [3], who showed increased female germ cell apoptosis in mice from 20.5 to 22.5 dpc.

The proportion of P^-^T^+ ^oocytes in pachytene was low except on 16 dpc (figure 5). P^-^T^+ ^oocytes are either healthy, with physiological DNA breaks due to meiotic chromosome activities [48,49] or active RNA synthesis [20] or at very late stages of apoptosis, with migration of cleaved PARP-1 from the nucleus to the cytoplasm [50] and consumption of NAD^+ ^in the cytoplasm [51] to replenish ADP-ribose. Since their elements are intact, and pachytene oocytes are increasing in number at this point, the latter seems unlikely. The nature of the TUNEL positivity in PARP-1 negative pachytene oocytes could possibly relate to recombination activities at this time [52], however, in that case, it is unclear why it affects only a proportion of pachytene oocytes.

Previous studies have noted abnormal appearing pachytene oocytes using histological methods and have concluded that pachytene is a major point in meiosis at which oocyte elimination may occur [2,25,53] as is the case in males [54]. However, this was challenged by McClellan et al [35] who used a combination of histological and spreading methods to show a continual loss of oocytes throughout meiotic prophase I. In our study, pachytene oocytes were no more likely than other stages of meiotic prophase to exhibit fragmented or compressed elements (Figure 2), or staining for apoptotic markers. Our data therefore do not support the contention of stage-specific apoptosis in prenatal mouse oocytes.

## Conclusion

In summary, fragmented axial elements, demonstrated by COR 1 staining, are not necessarily indicative of oocyte apoptosis, however, compressed elements in poorly-spread nuclei may be associated with apoptosis. Approximately 10–50% of oocytes at all stages stained positive for cleaved PARP-1, an apoptosis marker. These conclusions have major implications for the interpretation of data arising from oocyte spreading techniques. In particular, our data pose a significant challenge to the currently widespread assumption that fragmented axial elements are evidence of oocyte degeneration. It is possible that such oocytes may be undergoing a cell death process unrelated to PARP-1. However, PARP-1 dependent apoptosis is clearly a major contributory pathway for prenatal oocyte loss during meiotic prophase I because a major fraction of this oocyte population does stain for PARP-1, regardless of the appearance of axial elements. Future research should address which other apoptotic pathways are involved, the upstream events leading to a cell death decision, and how they relate to the control and progress of meiotic prophase I.

Our results also provide strong support for the work of others challenging the concept of stage-specific oocyte demise during meiotic prophase I. The proportions of P^+^T^+ ^oocytes during zygotene on 15.5 dpc, pachytene from 16 to 17 dpc and diplotene from 19 dpc onwards suggests that there are several stages throughout first meiotic prophase when apoptosis may occur. However, diplotene seems to be when the majority of oocytes are depleted via apoptosis, particularly at birth or shortly afterwards. Some oocytes in zygotene and pachytene, which lack evidence of apoptosis, may persist for at least 2 days postnatally. Genetic effects upon the rate of meiotic prophase I and the numbers of oocytes that will survive beyond the prenatal period are currently poorly understood, but may explain some of the discrepancies noted between our work and that of others.

Prenatal apoptosis and events in meiotic prophase I are well known to impact upon many later aspects of oogenesis and fertility, and thus deserve thorough investigation. The present data add to the evidence detailing oocyte apoptosis throughout meiotic prophase I, providing much-needed information and challenging the validity of TUNEL for studies of apoptosis in oocytes.

## Methods

### Mice

The mice were kept under Home Office licence, housed at 23°C with 12:12 hours of light:dark, and fed *ad libitium*. Female B6CBF1 mice aged from 6 weeks to 6 months old were caged with a male overnight for one night only, to ensure accuracy of dating their pregnancy.

### Collection and preparation of ovarian tissues

The first day of observing the copulation plug was counted as day zero. Pregnant females were sacrificed in a CO_2 _chamber on specific days *post-coitum *(dpc) in the morning (14.5, 15.5 dpc) or evening (16, 17, 18, 19, 20, 21 dpc). Ovaries were dissected from female fetuses between the ages of 14.5 and 18 dpc. Some pregnant mice were allowed to deliver their litter, which occurred on the morning of day 19. Neonates were sacrificed using CO_2 _at 19 to 21 dpc and neonatal ovaries were collected.

Both ovaries from at least three B6CBF2 mouse fetuses/neonates at each time point were placed in protein-free Ham's F10 medium (Sigma, UK). The ovaries of each fetus/neonate were kept separately from those of others. The entire process of ovary isolation lasted ~20 minutes. To obtain microspread oocyte preparations, whole ovaries were macerated in protein-free Ham's F10 medium on an ethanol-cleaned glass slide and further prepared as described below. One ovary was used per slide.

### Determination of stages of meiotic prophase I and detection of apoptosis

Cleaved PARP-1 and COR1 were detected simultaneously on micro-spreads of mouse ovaries, followed subsequently by TUNEL labelling of DNA breaks.

#### 1) Co-detection of COR1 and cleaved PARP-1

COR1 protein is present on axial elements of the synaptonemal complexes between homologous chromosomes during all stages of meiotic prophase I [55]. Therefore the long-term presence of COR1 on the core elements of chromosomes during meiosis makes it a useful germ cell marker. Fluorescent highlighting of COR1 protein demonstrates the arrangement of chromosomal pairing and hence the stage of meiotic prophase I. Anti-COR1 antibody recognizes short segments of chromosomal core elements at the leptotene stage and fully formed elements at the pachytene stage [56]. COR1 protein was identified using polyclonal mouse anti-hamster COR1 antibody (a kind gift from Peter Moens, York University, Toronto, Canada).

PARP-1 is activated by binding to DNA strand breaks, where it catalyses the transfer of ADP-ribose from NAD^+ ^to certain proteins involved in chromatin architecture or DNA metabolism including PARP-1 itself [57]. PARP-1 is proteolysed during apoptosis, converting from a 116 kDa form to fragments of 89 kDa (C-terminal fragment) and 24 kDa (N-terminal fragment) [58]. The presence of cleaved PARP-1 indicates an incapacity to repair DNA, which is considered a marker of apoptosis [59] and can be revealed by specific antibodies.

Briefly, dispersed ovarian cells were treated with 3 drops of 3% sucrose hypotonic solution for 30 minutes at room temperature. The spreads were fixed with 10 drops of 1% ultra pure formaldehyde (TAAB, Aldermaston, UK) containing 1% SDS, pH: 8.0 for 25 minutes at room temperature. After fixation all slides were washed for 5 minutes with 0.5% triton in PBS and then twice for 10 minutes with 0.1% triton in PBS (PBT). All slides were incubated with 5% normal goat serum (Sigma) in PBT for 45 minutes at room temperature, to prevent non-specific binding. Primary anti-COR1 antibody at a concentration of 1:1000 in PBT and primary anti-cleaved PARP-1 antibody (rabbit anti-mouse, polyclonal antibody, Cell Signalling, USA) at a concentration of 1:50 in PBT, were applied simultaneously. Slides were placed in a moist chamber at 4°C overnight.

All secondary antibodies were used at a concentration of 1:200 in PBT. Texas Red conjugated goat anti-mouse antibody (Vector laboratories, UK) was applied for 30 minutes in the dark at 37°C to visualise COR1 followed by three further washes in PBT for 5 minutes. To visualise cleaved PARP-1, goat anti-rabbit biotinylated IgG (L+H))(Vector laboratories) was applied for 30 minutes at 37°C followed by 3 washes in PBT of 10 minutes each. Then a combination of Texas Red goat anti-mouse and anti-avidin AMCA [7-amino-4-methylcoumarin-3-acetic acid (Vector laboratories)] was applied and incubated for 30 minutes at 37°C. Afterwards the slides were washed 3 times in PBT, 10 minutes each. Finally the slides were mounted with Vectashield mounting medium for fluorescence without DAPI (Vector Laboratories). All slides were viewed directly under fluorescence microscopy (Axioskop, Carl Ziess) to detect individual oocytes (highlighted by COR1 staining), noting the presence or absence of cleaved PARP-1 indicated by blue staining of nuclei where the axial elements were highlighted with COR1. Fluorescence microscope images were recorded via a cooled charged-coupled device (CCD) camera and Vysis QUIPS with Smart capture software (Digital Scientific).

Negative controls lacking primary and/or secondary antibodies were performed at the same time as test slides to confirm no cross reactivity between anti-COR1 and anti-cleaved PARP-1 primary and secondary antibodies.

All the slides were then further processed for detection of DNA fragmentation using TUNEL with a direct fluorescent method as described below.

### Criteria for classification of oocytes according to COR1 staining

Oocytes in definitive stages of meiotic prophase I were distinguished using criteria set out by Barlow and Hultén [28] and Hartshorne et al [9] with more detail regarding the integrity (intact/fragmented) of the axial elements [11]. During preleptotene, the nuclei of the oocytes accumulated COR1 protein but displayed only very short segments of proteinaceous backbone. During the leptotene stage, staining of COR 1 protein was apparent on the proteinaceous backbone forming along each chromatid pair. Leptotene oocytes were considered normal unless an unusual assembly of COR1 on axial elements was observed. During the zygotene stage, the staining was denser and homologous chromosomes had begun to align with one another according to classification by Wallace and Hultén [26] and Bojko [27]. At the pachytene stage, homologous chromosomes were fully synapsed along their entire length, forming 20 distinct bivalents apparent as compressed, shortened structures [review [60]]. During the diplotene stage, homologous chromosomes had become separated by repulsion and started to desynapse. Crossover sites were apparent holding the homologous chromosomes together by chiasmata [see [60]]. Distinction between the zygotene and diplotene stages was possible since the axial elements in zygotene, with longer lengths and opened non-synapsed ends, appeared different from those in the diplotene stage that are rather shorter in length and very dense with forklike desynapsed ends.

The total number of cells having COR1 staining on axial elements was counted on each slide (one ovary per slide). The axial elements of homologous chromosomes in oocytes were classified as intact when there was continuous staining of COR1, fragmented when there were discontinuities or large gaps in COR1 staining, compressed when the oocyte nucleus was not well spread, and degenerated when scattered staining was observed but there was no clear structure to the axial elements. Preleptotene and leptotene oocytes were excluded from the analysis on fragmentation of elements, since their elements are, by definition, fragmented at this stage of meiotic prophase I. Although incomplete elements were present in zygotene oocytes, they were distinguishable from fragmented elements by the extent to which they were linear and partially paired. Oocytes were classified as abnormal when the appearance of their intact elements did not match with the criteria of axial elements in any stage of meiotic prophase I, as described above, based upon published descriptions [9,26-28,46]. Some oocytes stained with COR1, yet could not be staged using the criteria above. These represented 13.7% of the total.

#### 2) TUNEL labelling

DNA cleavage was detected by TUNEL using the Apop Tag Fluorescein Direct In Situ Apoptosis Detection Kit (Intergen, USA) that enzymatically labels free 3'-OH ends with fluorescein nucleotides. This technique is used in many applications to detect apoptosis where chromatin condensation has begun and DNA breaks are occurring. We applied it here to study DNA breaks on the same micro-spread oocytes that had already been assessed using antibodies to COR1 and cleaved PARP-1. All washing and incubation processes were performed in the dark in order to preserve the fluorescent markers. After removal of residual immersion oil and glass cover slips, the slides were re-stained with Texas Red-labelled goat anti-mouse antibody for 30 minutes in the dark at 37°C, to ensure that the axial elements would remain completely visible. Slides were washed in PBT, three times for 10 minutes and then processed according to the manufacturer's protocol for TUNEL. Positive control slides were treated with 0.9 μg/ml DNAse 1 in DN buffer for 10 minutes at room temperature. Finally slides were mounted with Vectashield mounting medium for fluorescence without DAPI (Vector Laboratories). These slides were either viewed immediately or stored at -20°C.

The same oocytes previously examined for COR1 and cleaved PARP-1 staining were located and assessed for TUNEL labelling and their images were captured. During these assessments oocytes were categorised into four different groups (P^+^T^+^, P^+^T^-^, P^-^T^+^, P^-^T^-^) according to being cleaved PARP-1 positive (P^+^) or negative (P^-^) and TUNEL positive (T^+^) or negative (T^-^).

All oocytes in which COR1 staining was identified were examined, including those which appeared to be in the process of degeneration, since the features of such oocytes were of particular relevance to our aims.

### Statistical analysis

The distribution of oocytes at different stages of first meiotic prophase in fetal ovaries was analysed by ordinal regression after logit transformation of the data. Within each stage of meiotic prophase I, each oocyte was classified according to its positive or negative staining for cleaved PARP-1 and TUNEL, and analysed by ordinal regression as before. Results were back transformed to obtain the proportions of oocytes at each stage of meiosis in each category of cleaved PARP-1 and TUNEL labelling according to their axial element integrity (intact, fragmented or compressed). Unstaged oocytes were omitted from the analyses of cleaved PARP-1 and TUNEL staining at different stages of development. Compressed oocytes where the stage of meiotic prophase I could be determined were included with intact oocytes for comparisons of intact and fragmented elements in terms of cleaved PARP-1 and TUNEL staining.

## Authors' contributions

FG designed the experiments, developed the method, carried out the collection and processing of ovaries, including immunocytochemistry, image analysis, collation and analysis of data. CGG carried out statistical analyses. GMH conceived of the study, participated in its design and coordination and supervised FG in its conduct, compilation, interpretation and presentation.
